# Mitochondrial Dysfunction and Chronic Inflammation in Polycystic Ovary Syndrome

**DOI:** 10.3390/ijms22083923

**Published:** 2021-04-10

**Authors:** Siarhei A. Dabravolski, Nikita G. Nikiforov, Ali H. Eid, Ludmila V. Nedosugova, Antonina V. Starodubova, Tatyana V. Popkova, Evgeny E. Bezsonov, Alexander N. Orekhov

**Affiliations:** 1 Department of Clinical Diagnostics, Vitebsk State Academy of Veterinary Medicine [UO VGAVM], 7/11 Dovatora str., 210026 Vitebsk, Belarus; 2 Center of Collective Usage, Institute of Gene Biology, Russian Academy of Sciences, 34/5 Vavilova Street, 119334 Moscow, Russia; nikiforov.mipt@googlemail.com; 3 Laboratory of Medical Genetics, Institute of Experimental Cardiology, National Medical Research Center of Cardiology, 121552 Moscow, Russia; 4 Laboratory of Cellular and Molecular Pathology of Cardiovascular System, Institute of Human Morphology, 3 Tsyurupa Street, 117418 Moscow, Russia; evgeny.bezsonov@gmail.com (E.E.B.); a.h.opexob@gmail.com (A.N.O.); 5 Department of Basic Medical Sciences, College of Medicine, QU Health, Qatar University, Doha 2713, Qatar; ali_eid@hotmail.com; 6 Biomedical and Pharmaceutical Research Unit, QU Health, Qatar University, Doha 2713, Qatar; 7 Department of Pharmacology and Toxicology, Faculty of Medicine, American University of Beirut, Beirut P.O. Box 11-0236, Lebanon; 8 Federal State Autonomous Educational Institution of Higher Education, I. M. Sechenov First Moscow State Medical University (Sechenov University), 8/2 Trubenskaya Street, 119991 Moscow, Russia; profmila@mail.ru; 9 Federal Research Centre for Nutrition, Biotechnology and Food Safety, 2/14 Ustinsky Passage, 109240 Moscow, Russia; avs.ion@yandex.ru; 10 Pirogov Russian National Research Medical University, 1 Ostrovitianov Street, 117997 Moscow, Russia; 11 V.A. Nasonova Institute of Rheumatology, 34A Kashirskoye Shosse, 115522 Moscow, Russia; popkovatv@mail.ru; 12 Laboratory of Angiopathology, The Institute of General Pathology and Pathophysiology, 8 Baltiyskaya Street, 125315 Moscow, Russia

**Keywords:** polycystic ovarian syndrome, insulin resistance, chronic inflammation, oxidative stress, mitochondrial mutations

## Abstract

Polycystic ovarian syndrome (PCOS) is the most common endocrine–metabolic disorder affecting a vast population worldwide; it is linked with anovulation, mitochondrial dysfunctions and hormonal disbalance. Mutations in mtDNA have been identified in PCOS patients and likely play an important role in PCOS aetiology and pathogenesis; however, their causative role in PCOS development requires further investigation. As a low-grade chronic inflammation disease, PCOS patients have permanently elevated levels of inflammatory markers (TNF-α, CRP, IL-6, IL-8, IL-18). In this review, we summarise recent data regarding the role of mtDNA mutations and mitochondrial malfunctions in PCOS pathogenesis. Furthermore, we discuss recent papers dedicated to the identification of novel biomarkers for early PCOS diagnosis. Finally, traditional and new mitochondria-targeted treatments are discussed. This review intends to emphasise the key role of oxidative stress and chronic inflammation in PCOS pathogenesis; however, the exact molecular mechanism is mostly unknown and requires further investigation.

## 1. Introduction

Stein–Leventhal syndrome, commonly known as polycystic ovarian syndrome (PCOS), is a complex, multifaceted endocrine disease with a global prevalence among women of reproductive age. PCOS is the most probable cause of anovulation-mediated infertility, striking about 2–26% of women of the 18–44 age group [1,2]. PCOS is considered a high-risk factor for several metabolic complications, such as MetS (metabolic syndrome), CVD (cardiovascular disease), T2DM (type 2 diabetes mellitus), and IR (insulin resistance), with a recent noticeable increase in cases of endometrial cancer [3]. Notwithstanding the decades of intensive research, the exact molecular mechanism of PCOS pathogenesis is still obscure. PCOS develops as a system disease connected to genetic and epigenetic changes that vary among different populations and family lineages [4,5]. PCOS diagnosis relies on the so-called “Rotterdam criteria”, defined in 2003 [6], where oligo- and/or anovulation, surplus androgen activity and polycystic ovaries are the main criteria. The Rotterdam criteria are regularly revised and should be assisted by several other genetic and molecular analysis [7]. However, PCOS is a highly heterogenic syndrome, with variable manifestations in different ethnic and age groups [8], so different cut-offs should be used for MBI, IR, hirsutism and other clinical signs [9]. Obesity, for example, is often observed in PCOS patients and known as an accentuating factor [10], albeit, for Asian ethnic groups, the connection between PCOS and obesity is less pronounced [11,12].

Mitochondria are the central player in energy production but also the main source of cellular ROS (reactive oxygen species), leading to OS (oxidative stress) damage. Due to this reason, mitochondrial abnormalities often have organism-wide manifestation and result in different metabolic disorders [13]. Nowadays, mitochondria-generated OS and chronic inflammation are recognised as the central causative factor of PCOS aetiology and associated with HG (hyperglycemia), anovulation, hyperandrogenemia and IR [14,15]. Additionally, coagulation disorder, NAFLD (nonalcoholic fatty liver disease) and atherogenic dyslipidemia have been often diagnosed in PSOC patients [16,17,18].

MiRNAs [19], mutations and SNPs of nuclear-encoded genes (such as *FSHR*, *LHCGR*, *DENND1A*, *THADA* and others) have been linked to PCOS development and pathogenesis [20,21]. However, detailed characterisation of the mitochondrial DNA mutations linked to PCOS is scarce; available data is narrowed to small ethnic groups. Comprehension of the role of mtDNA (mitochondrial DNA) mutations in PCOS pathogenesis and aetiology would facilitate the elaboration of better diagnostic tools and specific treatments. 

### PCOS as a Low-Grade Chronic Inflammation Disease

It is known that in up to 80% of patients, PCOS is followed by IR and obesity, which, altogether with other symptoms such as hyperandrogenism and hyperinsulinemia, can reinforce each other [22,23]. Furthermore, PCOS is known for its chronic inflammation status, delivered mainly by surplus adipose tissue [24]. Recent studies have identified elevated levels of several proinflammatory cytokines: TNF-α (tumour necrosis factor-alpha), CRP (C reactive protein), IL-6 (interleukin), IL-8, and IL-18. In addition to that, higher IR and lower total oxidant status have been found in PCOS patients [25,26,27,28]. Additionally, PCOS is consistent with high BMI (body mass index), WBC (white blood cell) and androgen concentration, suggesting that inflammation can be mediated through increased androgens [29]. The levels of adipokine omentin-1 [30] that are released by adipose tissue and mediated IR are lower in PCOS patients [31,32]. Additionally, several studies have identified SNPs (single-nucleotide polymorphisms) in genes of proinflammatory cytokines linked with PCOS, thus suggesting a genotype-specific predisposition to PCOS: TNF-α and IL-6 [33], IL-10 [34], IL-17A and IL-32 [35]. The level of the hormone leptin, mostly produced by adipose tissue, is also elevated in PCOS patients; it upregulates INF-γ (interferon-gamma) and IL-6 production and binds with IR [36]. In total, these data represent a crucial connection between the metabolic manifestation of PCOS and chronic inflammation.

In this review, we will summarise recent achievements in understanding the role of mitochondrial mutations and chronic inflammation in PCOS pathogenesis and novel targets in PCOS therapy and diagnosis.

## 2. Role of Mitochondria in PCOS

Mitochondria are organelles responsible for energy generation via OXPHOS (oxidative phosphorylation), implicating many nuclear-encoded proteins and 37 of the most crucial proteins and RNAs encoded by mitochondrial DNA—2 rRNA (ribosomal RNAs; 16S and 12S), 22 tRNAs (transport RNAs) and 13 proteins of the OXPHOS system (complexes I, III–V). The 38th protein—humanin (encoded by 16S rRNA)—has protective activities against apoptosis-mediated diseases [37]. All other proteins required for the mitochondria regulation, maintenance and repair are encoded by the nuclear genome [38].

Two crucial distinctions from nuclear DNA are maternal inheritance and a variable number of mtDNA copies per cell. The latter feature makes possible the presence of different genotypes in the same cell/tissue, termed heteroplasmy; accordingly, the presence of only one allele is called homoplasmy. Mostly, pathogenic mutations are heteroplasmic, and the level of heteroplasmy correlates with the manifested phenotype [39]. Dysfunction in any of the main cellular functions of mitochondria (OXPHOS-mediated ATP synthesis, ROS generation and regulation, Ca^2+^ buffering, cell cycle and signalling control) can result in disease development. It is known that ROS are toxic byproducts that are able to damage mitochondrial and nuclear DNA, lipids, and proteins and cause developmental disorders and diseases [40,41]. Similarly, proper Ca^2+^ regulation is required to control membrane permeabilisation and prevent the release of toxic components of inner mitochondrial content to the cytosol [42]. Additionally, Ca^2+^ is the major agent in ER-mitochondrial communication, necessary for proper cellular signalling, membrane dynamics and lipid transfer, and violation of this connection may cause disease development [43]. Due to proximity to the source of ROS generation and the lack of histones and DNA repair mechanisms, mtDNA is more assailable to OS damage and has a higher chance of acquiring mutation [44]. Another marker of healthy mitochondria, associated with many developmental disorders and age-related diseases, is mtDNA copy number [45]. The role of mtDNA copy number in PCOS is rather conflicting. Different groups have reported positive, negative or no association between mtDNA copy number and PCOS [46,47,48]. This aspect of PCOS pathogenesis requires further investigation, with wider racial and ethnic representation of patient groups and a deeper examination of the associated symptoms and morbidities.

ROS are widely recognised as a harmful agent responsible for the aetiology and progression of hundreds of human diseases (including cancer, atherosclerosis, CVD, PCOS, DM (diabetes mellitus) and many others) [49]. In the case of PCOS, generated ROS are the main pathogenesis factor; they is directly linked to mtDNA replication and mitochondria OXPHOS efficiency. ROS damage biological molecules when the levels of antioxidant enzymes are lower than necessary. This suggestion is proven by circulating markers of ROS (MDA, SOD (superoxide dismutase) and glutathione peroxidase), which are significantly higher in PCOS patients [15]. In addition to their direct role in the pathogenesis of the disease, ROS also act as redox messengers, participating in many cellular signalling pathways, regulating cell growth, differentiation, proliferation and apoptosis [50]. Several studies have proven a tight bond between increased ROS, PCOS development [51] and other associated conditions like hyperandrogenism [52] and MetS [53]. Elevated ROS are damaging DNA-repair proteins that cause further mutations, OXPHOS malfunction and the production of more ROS, making the vicious cycle complete [54].

### Identification and Analysis of Pathogenic mtDNA Mutations in PCOS Patients

**Figure 1:**
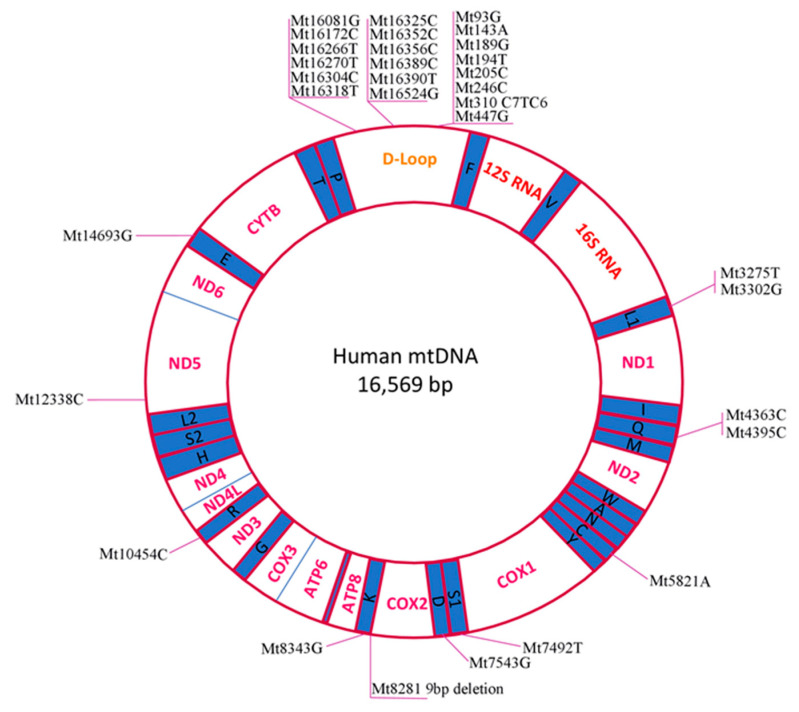
Simplified map of the human mitochondrial genome. The mtDNA mutations associated with PCOS, listed in Table 1, are marked.

We have summarised the mtDNA mutations identified in PCOS patients (Table 1). The table combines data from case studies (one patient/family) and wide population analyses. Listed mutations were identified backwards from PCOS-diagnosed patients (Rotterdam-criteria-based), and healthy people were used as a control. Identified mutations have been marked on the human mitochondria genetic map (Figure 1).

In total, 33 PCOS-related mtDNA mutations have been found. Twelve mutations have been found in tRNA genes and only 2 in OXPHOS system components. The majority of the identified mutations (20) were identified in the D-loop regulatory region, suggesting it as a hot-spot for PCOS-related mtDNA mutations. 

Despite several papers having proposed mtDNA mutations as a causative agent for PCOS development, there are some arguments against such a causative relation. The main arguments are as follows: (1) the homoplasmic nature of the identified mutations are not associated with other severe clinical symptoms usual for mtDNA mutations that lead to premature ageing and death; (2) some mutations are also present in healthy controls; (3) skeletal muscle functions are not affected [63,64]. Additionally, we have to note that the obtained results have originated from only two ethnic groups (Chinese Han and South Indian), so we cannot rule out selection bias. It is possible that the observed mutations are an example of natural mtDNA variation because wide-population mtDNA analysis of those ethnic groups is still missing. The next crucial point is the lack of standardisation in phenotype description (levels of hormones, degree of oligo/anovulation, and related symptoms like DM, IR, MetS, hypertension, CVD and others) [65].

Mitochondria are crucial organelle for intracellular redox metabolism; they produce ROS and release other intermediates of the TCA (tricarboxylic acid) cycle, which are normally neutralised by the antioxidant defence system. While we cannot directly link the causal relationship between mtDNA mutations and PCOS, there is a clear confirmation of disordered mitochondrial functions in PCOS patients. Mitochondrial structure, dynamics, biogenesis and MMP (mitochondrial membrane potential) are disturbed in PCOS patients [66]. The mitophagy process required to remove abnormal and aged parts of mitochondria and damaged mitochondrial proteins is dysregulated in PCOS patients. As a result, such mitochondria have a pathological oxidative status. In particular, the level of MDA (malondialdehyde) is increased, while the levels of ROS-protecting molecules (glutathione, SOD and catalase) are decreased, representing a high oxidative stress index [66,67,68].

In conclusion, these results suggest that mutations in mtDNA, especially within the D-loop region, could be linked with PCOS. On the other hand, the causative role of those mutations in PCOS aetiology is still a matter of further research. For proper characterisation and conformation of the pathogenicity of mtDNA mutations, all patients need to be traced for 2–3 generations on their history of family diseases and undergo full multiorgan diagnosis and long-term follow-up clinical observation.

## 3. Novel Markers for PCOS Diagnosis

PCOS diagnosis relies on the well-established Rotterdam criteria [6], where ultrasound is used for ovary examination and laboratory methods are applied to measure sex hormones (primarily, serum LH and testosterone). Additionally, other comorbidities such as MetS, DM, CVD and cancer, often associated with PCOS, should also be checked [1]. Additionally, recent research has suggested using more markers for the early prediction of PCOS. Thus, levels of DHEA (dehydroepiandrosterone), insulin, HbA1c (glycosylated haemoglobin) and LDL-c (low-density lipoprotein-cholesterol) could be used for the early diagnosis of PCOS [69]. Xenin, an ancient regulatory peptide [70] known as a regulator of insulin and glucagon secretion (thereby an important therapeutic target in DM and obesity [71]), was also shown to be a factor of PCOS pathogenesis [72].

Many research projects have proposed that the imbalanced immune system cells in PCOS patients are due to the chronic inflammation nature of PCOS. Earlier, it was found that Th1 cells and the Th1/Th2 ratio were higher in PCOS patients [73], and the authors suggested a connection with abdominal obesity [74]. It was shown that three proteins (phosphatidylethanolamine-binding protein 1, proteasome activator complex subunit 1 and triosephosphate isomerase) responsible for increased glycolysis are overexpressed in the Th cells of PCOS patients [75]. NK (natural killer) cells, another type of lymphocyte crucial for an adaptive immune response, are also increased in PCOS patients and can serve as a diagnostic factor [76].

Glycoproteins are key players in the inflammation process. It is known that during inflammation, some proteins undergo structural changes and glycan chains modifications; thus, glycoprotein profiles can serve as an inflammation marker [77]. GlycA and GlycB biomarkers of inflammation, IR, insulin secretion and obesity [78,79] have also been recently linked to PCOS [80].

However, we should note that the analysis of metabolomic and chronic inflammation parameters should be done with care because PCOS patients have frequent body composition changes and different diet and feeding behaviour, which could influence the obtained results [81].

## 4. PCOS Treatments: Classical and Modern Mitochondria–Oriented Approaches

### 4.1. Mouse Studies

For decades, animal models have been used to study human PCOS. Despite the obvious difference in reproductive physiology between human and rodents, animal models provide a great contribution to PCOS research. As a main disadvantage of the rodents as a PCOS model, we could mention the absence of natural PCOS-like features, such as hormonal disbalance and, primarily, hyperandrogenism. Thus, model animals have induced PCOS achieved by direct hormonal intervention (testosterone propionate, dihydrotestosterone, estradiol valerate, human chorionic gonadotropin, LH, AMH (anti-Müllerian hormone), GnRH (gonadotropin-releasing hormone)), indirect hormonal perturbation (natural and genetically engineered mutations), and metabolic dysfunctions (diet, environmentally or chemically induced). Due to these limitations, research with animal models requires carefully considered control/treatment and reproducible methods [82].

Metformin. Current experimental PCOS treatments are based on the combination of well-known DM drugs, general health-improving compounds and specific mitochondria-targeted medications. Metformin is a well-known drug in DM treatment; the molecular mechanism of its activity is based on the inhibition of mitochondrial glycerophosphate dehydrogenase in the liver. Metformin helps to reduce fasting insulin concentrations and stabilise blood pressure and LDL-c levels [83]. The exact molecular mechanism of metformin’s action on ovarian cells relies on the phosphorylation of AMPK (5’ AMP-activated protein kinase), a crucial player in energy homeostasis that activates glucose and lipid uptake and oxidation [84]. Additionally, metformin has a long history of use as first-line PCOS treatment [85]. Nowadays, metformin is combined with other drugs, primarily with gut microbiota, improving probiotic inulin (fructose polysaccharide). Thus, in DHEA and high-fat diet-induced PCOS mice models, inulin and metformin treatments have resulted in lower levels of inflammation markers (TNF-α, IL-6 and IL-17A) and lower amounts of pathogenic bacteria *Helicobacter* and *Parasutterella* in gut microbiota [86].

Another popular PCOS treatment (metformin and Diane-35 (cyproterone acetate and ethinyl estradiol)) has been shown to normalise ATP and lactate levels and increase expression levels of glycolysis-related rate-limiting enzymes (PKM2 (pyruvate kinase isozyme M2 isoform) and LDH-A) and NAD^+^-dependent deacetylase SIRT1 (silent information regulator 1) [87]. SIRT1 maintains blood glucose levels via the regulation of gluconeogenic/glycolytic pathways in the liver and improves oxidative stress-related insulin resistance [88]. IR is the main target in the combined application of metformin and Exenatide (synthetic GLP-1 receptor agonists), a clinically used T2DM treatment; as a result, body weight and levels of glucose, IR and androgens decrease, while the expression of SIRT1 and AMPKα increase [89].

Metformin has a synergic effect with bee pollen, decreasing levels of TNF-α, NO (nitrogen oxide) and cancer marker Ki-67 [90]. Similarly, metformin has a synergic effect with flutamide, an androgen receptor antagonist used in hyperandrogenemia and dyslipidemia treatments. Thus, a combined treatment reduces the intestinal secretion of ApoB48 (apolipoprotein B) and the plasma level of androgens and ameliorates insulin signalling [91]. As ApoB48 is known for its role in T2DM and its high atherogenic potential [92], combined metformin–flutamide treatment is preferable for the CVD high-risk group of PCOS patients. Metformin administered with natural plant phenol and phytoalexin resveratrol resulted in decreased levels of steroid hormones (LH (luteinizing hormone), FSH (follicle-stimulating hormone), testosterone), AMH, and TNF-α and increased expressions of SIRT1 and AMPK in DHEA-induced PCOS rats [93].

Hormone melatonin. Melatonin dysregulation is a crucial part of PCOS pathogenesis. It is known that melatonin is a free radical scavenger and circadian rhythm regulator that can improve metabolic functions and ameliorate PCOS symptoms [94]. Thus, the application of melatonin results in the normalisation of melatonin, cytokine (MT1, MT2 and IL-2R, IL-6R, respectively) and Er-α (estrogen) receptors, lowering levels of IL-6 and TNF-α. The described mechanism implies regulatory crosstalk between melatonin receptors (MT1R/MT2R) and cytokinin receptors (IL-2R, IL-6R), modulating Er-α expression [95].

Natural compounds. Natural compounds have high potential in PCOS therapy. Flavonoid baicalin (isolated from *Scutellaria baicalensis*) has been shown to decrease levels of testosterone, LH, progesterone and estradiol, and several proinflammatory cytokines (IL-1β, IL-18 and TNFα) and normalise IR in PCOS-induced rats [96]. Similarly, flavonoids recovered from *Nervilia Fordii* decrease the levels of IL-6 and hormones LH, testosterone and insulin and increase the serum level of FSH [97]. Plant-delivered alkaloid berberine has been shown to reduce levels of IR and testosterone in PCOS-induced rats. Additionally, beneficial effects were observed for the expression of IL-1, IL-6, TNF-α and NF-kB, also suggesting anti-inflammatory activities [98]. There are many reports providing a positive effect on PCOS and related morbidities after treatment with traditional herbal medicine [99,100]; however, since the exact active compounds are not specified, we will not cover them in this review and interested readers can follow the cited publications.

#### Mitochondria-Targeted Therapy

Vitamin D. Mitochondrial dysfunction plays a crucial role in PCOS development [101]. Thus, significant efforts have been made to normalise mitochondria performance and ameliorate PCOS symptoms. Vitamin D is one of the first supplementation medicines used to improve the blood lipid status of PCOS patients [102]. However, the results have been contradictory, suggesting a rather minor influence in the reduction of total cholesterol, while the most harmful fraction, HDL-c (high-density lipoprotein cholesterol), was not affected [103,104].

Nevertheless, it is known that vitamin D metabolism in PCOS is disrupted and, most probably, contributes to PCOS pathogenesis [105]. Thus, it is not surprising that vitamin D has a direct effect on mitochondria; it improves biogenesis and antiapoptotic parameters and upregulates the expression of antioxidants (SOD, catalase and glutathione peroxidase), which lead to a decrease in ROS levels [106]. Vitamin D treatment stimulates the expression of the TFAM (mitochondrial transcription factor A) gene, the main nuclear regulator of mitochondria biogenesis, leading to a higher copy number of mtDNA and improved membrane integrity [107]. Vitamin D supplementation improved ovary and uterus morphology and decreased weight and obesity levels in DHEA-induced PCOS rat models [108]. Additionally, vitamin D regulates steroidogenesis by reducing the expression of steroidogenic enzymes and reduces the production of progesterone and 17B-estradiol [109]. Similar results were obtained for vitamin D treatment combined with antioxidant MitoQ_10_, resulting in the normalisation of hormonal status (FSH, LH, estradiol and progesterone) and the level of OS markers (SOD and MDA) [110]. Administration of MitoQ_10_ alone improved mitochondrial function through the regulation of the programmed cell-death mechanism. Thus, the expression of apoptosis-related proteins cytochrome c and BAX (B-cell lymphoma-2 (Bcl-2)-associated X protein) was decreased, whereas antiapoptotic Bcl-xL (B-cell lymphoma-extra large) increased after MitoQ10 treatment [111]. A similar effect on the apoptosis mechanism was achieved by sodium selenite treatment; in addition to the upregulation of antiapoptotic Bcl-xL and the downregulation of cell-death accelerator BAX proteins, the authors also observed improved lipid profiles in a letrozole-induced PCOS rat model [112].

### 4.2. Human Therapy

For decades, PCOS treatment has relied on diet intervention, lifestyle modification and hormone-normalising drugs. To date, physical exercise is the simplest way to mitigate inflammatory milieu in PCOS patients. Exercise increases levels of anti-inflammatory cytokines (IL-4, IL-10), decreases levels of the proinflammatory cytokine TNF-α and reduces the levels of phosphorylation of the main inflammatory response pathways IKKα/β/JNK [113]. Additionally, exercise is known to improve the levels of sex hormones (testosterone, follicle-stimulating hormone) and insulin sensitivity [114,115]. There are several known molecular mechanisms involved in the beneficial effects of exercise. The primary effect on insulin sensitivity and glucose metabolism relies on activation of the PI3K/AKT signal pathway and glucose transporter type 4, which allows muscles to absorb more glucose. Androgen status is improved through the inhibition of the 5α-reductase enzyme, which is known to metabolise testosterone. Additionally, exercise activates the mTOR signalling mechanism involved in the regulation of anabolic processes in muscle, improving insulin sensitivity [116,117,118].

There are several main targets in diet intervention: weight loss, improving liver function, blood glucose levels and lipid profiles, and ameliorating gut microbiota [119]. The main types of diet interventions are low-carbohydrate, calorie restriction, ketogenic, pre/probiotics prescription and mineral (chromium, zinc, calcium) supplementation [120,121].

Metformin. Metformin is the oldest insulin-lowering drug, well-known to improve hyperandrogenism, cause weight loss and ameliorate other PCOS-related abnormalities [122,123,124]. Metformin has been shown to reduce levels of proinflammatory cytokine IL-6 [125], acute phase inflammation and oxidative stress protein CRP [126]. Metformin administration results in a decreased level of leptin and an increased level of adiponectin in PCOS patients [127]. Both hormones—leptin, regulating energy balance, and adiponectin, regulating glucose and fatty acid metabolism—are mostly produced in adipose tissue and can provide synergistic activity [128]. Thus, metformin-mediated regulation of leptin/adiponectin levels is a promising way to reduce the risk of IR, T2DM and MetS in PCOS patients.

In combination with effective CVD medicine (atorvastatin), metformin has shown a solid reduction of adipose tissue proinflammatory state by lowering the levels of acylation-stimulating protein, IL-6 and MCP-1 (monocyte-chemoattractant-protein-1) and normalising the levels of testosterone, IR and CRP [129]. As suggested by in vitro study, metformin inhibits Er-α expression, with no effect on Er-β expression, and improves glycolysis and normalises mitochondrial function by increasing the expressions of TFAM, phosphofructokinase, lactate dehydrogenase A, PKM2 and cleaved caspase-3 [130].

The reduction in androstenedione, FSH, and IR and the increase in DHEA-S after metformin treatment can be explained by its lipidomic-profile-modulating activity. In particular, metformin decreases levels of sphingolipids, glycerophospholipids, and several lipoxidative species [131]. Ceramides are known to regulate testosterone and progesterone production and mediate IR, thus suggesting close sphingolipid–steroid hormone interaction [132].

It is known that glucose metabolism is dysregulated in PCOS patients. DPP4 (dipeptidyl peptidase-4), one of the key proteins in glucose homeostasis (regulating insulin secretion), is associated with several diseases and has increased activity in PCOS patients [133]. Sitagliptin is a DPP4 inhibitor, a promising drug in PCOS treatment [134]. Combined treatment “sitaformin” (sitagliptin/metformin) decreases HbA1c and glucose levels and improves pancreatic β-cell function and sensitivity to insulin [135].

Thiazolidinediones. Thiazolidinediones (TZDs) are a well-known family of drugs used to improve levels of lipids and glucose, mainly in T2DM (but they are also applied to nonalcoholic steatohepatitis, psoriasis, autism and other conditions) [136]. For PCOS treatment, TDZs were successfully used in combination with metformin [137] and other natural compounds (such as inositol and α-lipoic acid) [138], improving metabolic and endocrine parameters. However, TDZs had worsening weight gain that is considered a main negative side effect [139].

TDZs act by activating peroxisome proliferator-activated receptor gamma (PPAR-γ), which is localized mainly in adipose tissue and regulates several genes related to lipids, glucose metabolism and inflammation [140]. PPAR-γ increases insulin sensitivity via enhanced adiponectin release from adipocytes, reduces lipotoxicity and regulates cholesterol efflux and glucose uptake [141,142]. It is interesting that many natural compounds are able to bind directly with and activate PPAR-γ; such activity was shown for vitamin E and omega-3, carnitine, curcumin, chromium and melatonin [143,144,145,146], thus, suggesting (PPAR-γ) as a promising target for PCOS treatment.

Hormones. The hormone melatonin regulates behaviour and metabolic and immune functions and has antioxidant and ROS-scavenger properties, crucial for reproductive health [147]. A PCOS patient’s melatonin ameliorates mitochondrial functions by increasing the levels of SIRT1 and sirtuin protein and regulating mitochondrial functions via deacylation. The level of PINK1, the main mitochondrial quality control protein, on the contrary, was decreased [148]. Thus, melatonin administration in PCOS patients protects against mitochondrial injury in a SIRT1-dependent way.

Growth hormone (GH) has a long history of administration in cases of infertility and patients with disordered ovulation [149]. GH treatment reduces TOS (total oxidant status) and OSI (oxidative stress index) in PCOS patients’ oocytes, improves MMP and decreases apoptosis by >50% [150]. The exact molecular mechanism of such GH activity implies the activation of the PI3K (phosphatidylinositol 3-kinase) signalling pathway. The PI3K/Akt signalling pathway regulates cells proliferation, migration, survival, and metabolism and is involved in the development of many diseases. The PI3K/Akt pathway is involved in the regulation of proapoptosis genes through the phosphorylation of the FOXO transcription factor. It is known that the PI3K/Akt signalling pathway is dysregulated in PCOS patients [151]. GH treatment caused decreased levels of FOXO1, BAX and caspase3 and 9, while levels of PI3K, Akt and Bcl-2 were increased, thus reducing the apoptosis rate and alleviating mitochondrial dysfunction [152].

Natural compounds. Inositol is a promising medicine suggested for PCOS treatment; it modulates steroid and glucose metabolisms, improves IR status and acts like an antioxidant [153]. Inositol is also recommended for clinical treatments in combination with other antioxidants, for example, *α*-lipoic acid [154], to improve IR and oxidative stress status. Similarly, administration of α-linolenic acid normalises levels of plasma cytokines (IL-1β, IL-6, IL-10, IL-17A, TNF-α and MCP-1) and steroid hormones (LH, FSH, estrogen, progesterone and testosterone) and decreases IR [155]. As a promising insulin sensitiser, inositol acts via the activation of AMPK and sodium/myo-inositol transporter 1, which subsequently elevates GLUT-4 levels, increasing glucose uptake [156].

Inositols are effective and universal fertility-improving compounds that can be used for the treatment of obese patients without PCOS [157], lean PCOS patients without IR [158] and obese PCOS patients [159]; however, other research suggests that the inositol effect is IR-dependent [160].

The role of inositols in PCOS treatment has been covered in several recent reviews, to which we wish to redirect interested readers [161,162,163].

Salicylates are a well-known class of nonsteroidal anti-inflammatory medicines, inhibiting IκB-kinase β and preventing NF-κB activation, increasing insulin sensitivity and improving glycemic status in T2DM [164]. Treatment of PCOS patients with salicylates resulted in lower levels of ROS, NF-κB and TNF-α and stabilised androgens [165]. Nevertheless, salicylates should be administered with care due to the number of adverse effects, such as altered cardiorenal functions and hypoglycemia [166].

Administration of vitamin D, a well-known antioxidant, is also beneficial for PCOS patients. It has been shown that vitamin D reduces generated ROS and increases antioxidants (SOD and glutathione peroxidase) and steroid hormones (estrone and progesterone) synthesis [167]. The underlying molecular mechanism relies on the activation of enzymatic activities of two main estrogen biosynthesis enzymes (aromatase and 3β-Hydroxysteroid dehydrogenase/Δ^5–4^ isomerase); at the same time, reduced ROS will result in a lower apoptosis rate and higher cell viability, thus conforming to similar results obtained by another group [168].

In total, we could conclude that the application of well-known insulin-lowering and anti-inflammatory medicines are beneficial to overcoming PCOS symptoms. Similarly, the administration of vitamin D, alone and in combination with other antioxidants and natural compounds, could participate in several signalling mechanisms and provide a wide range of beneficial activities to improve mitochondrial function and reduce OS damage.

## 5. Long Noncoding RNAs—New Regulators of PCOS Inflammation

The first lncRNAs were discovered in 1990 [169] and considered “transcriptional noise”, or “junk” RNA without exact function [170]. Thanks to rapid progress in high-throughput sequencing technologies, about 60,000 lncRNAs have been identified in the human genome [171]. LncRNAs are a subclass of noncoding RNA transcripts, with an approximate length of around 200 nucleotides, that form unique three-dimensional structures and are involved in many regulatory mechanisms, namely, gene transcription, post-transcriptional (splicing/translation), epigenetic, DNA replication and others [172]. Recent research suggests that some lncRNAs can encode peptides, which act as crucial pathogenic agents of many diseases [173]. Additionally, lncRNAs play an important role in immune response and are involved in autoimmune disease development [174]. As a full-scale characterisation of lncRNAs is far beyond the scope of this review, we wish to redirect interested readers to the cited papers for further information. Recent research has discovered that 1583 lncRNAs are differently expressed in healthy and PCOS-diagnosed women [175]. Because of the complex and multifaceted involvement of lncRNAs in disease development and pathogenesis, we will further focus our discussion on inflammation-related lncRNAs.

LncRNA GAS5 (growth-arrest specific transcript 5) is known as a cancer-suppressor that regulates cell growth, survival, and proliferation and is also involved in cancer [176] and T2DM [177] aetiology. Recently, it was shown that GAS5 is involved in PCOS and the associated IR pathogenesis. Thus, PCOS patients with IR have decreased GAS5 expression and an increased concentration of IL-18 in serum [178]. Conversely, another study on PCOS patients and healthy controls revealed that GAS5 and IL-6 were upregulated in PCOS patients [179]. The described difference in GAS5 expression could be explained by the absence of the IR phenotype in the latter report [180].

Recent work supports a tight regulation between lncRNAs and cytokines. It was found that plasma levels of SRLR (sorafenib resistance-associated lncRNA in renal cell carcinoma) and IL-6 in PCOS patients were positively correlated and higher in comparison to healthy controls [181]. Interestingly, lncRNA SRLR also upregulates IL-6 transcription in RCC, where it is also associated with sorafenib resistance [182]. As a downstream partner, IL-6 activates STAT3 (signal transducer and activator of transcription 3), which is the key player in apoptosis and a crucial tumour progression marker [183]. This hypothesis is also supported in the rat DHEA-induced PCOS model system, where upregulated IL-6 and IL-11 have activated cell proliferation via the STAT3 signalling pathway [184].

LncRNA H19, the other known cancer progression biomarker [185], is also upregulated in PCOS patients and has been shown to interact with STAT3, synergistically regulating ovarian cells viability [186]. LncRNA SRA (steroid receptor RNA activator), known to be linked with several diseases and types of cancer [187], is upregulated in PCOS patients and linked to proinflammatory cytokine production (TNF-α, IL-1β and IL-6) and higher IR levels [188].

In total, these data suggest that lncRNAs are part of an important regulatory mechanism in PCOS aetiology, closely associated with apoptosis and inflammation pathways. Additionally, all the described lncRNAs are cancer biomarkers, which suggests a higher risk of cancer development for PCOS patients.

## 6. Conclusions

The data presented above suggest that mitochondrial dysfunction is involved in PCOS development and progression. Mitochondrial mutations, dysregulated mitophagy, decreased ATP production and released ROS also contribute to the related symptoms, primarily IR, MetS and obesity. Future research could concentrate on a better understanding of the causative role of nuclear and mitochondrial gene mutations and epigenetic, environmental and lifestyle factors on the aetiology of PCOS and the related symptoms. Current PCOS treatments rely on effective insulin-lowering, anti-inflammatory and symptom-targeted drugs and lifestyle and diet interventions. Novel methods imply the administration of synthetic and natural compounds to ameliorate mitochondrial function and reduce ROS levels and OS damage. As PCOS is the most common reproductive disease, it is crucial to find markers for early diagnosis and define preventive treatments. Since PCOS is recognised as a low-grade chronic inflammation disease, inflammation mediators and related signalling molecules could represent a novel target for therapeutic intervention. Recent research has helped to identify lncRNAs as a new regulatory mechanism involved in PCOS pathogenesis and, thus, a promising target for medical intervention. Hence, the potential use of new markers for early diagnosis, combined with target phenotype- and genotype-specific therapies, holds promising opportunities for PCOS patients.

## Figures and Tables

**Table 1:** List of mtDNA mutations identified in Polycystic ovarian syndrome (PCOS) patients.

Mutation	Gene	Other Notes	Year	
Mt8281 9 bp deletion	CO2/tRNA^Lys^	Chinese PCOS patients	2010	[55]
			2012	[56]
Mt3302G (A-to-G)	tRNA^Leu(UUR)^	One Chinese Han family case; T2DM and IR in family history, confirmed PCOS-IR in the proband	2016	[57]
Mt12338C (T-to-C)(M→T)	ND5	One Chinese Han patient, PCOS-IR; double mutation	2016	[58]
Mt7492T (C-to-T)	tRNA^Ser(UCN)^			
Mt3275T (C-to-T)	tRNA^Leu(UUR)^	One Chinese family, PCOS-MetS diagnosed; hypertension and T2DM in family history; triple mutation	2018	[59]
Mt4363C (T-to-C)	tRNA^Gln^			
Mt8343G (A-to-G)	tRNA^Lys^			
Mt3302G (A-to-G)	tRNA^Leu(UUR)^	Chinese PCOS patients with IR	2017	[60]
Mt3275A (C-to-A)				
Mt4363C (T-to-C)	tRNA^Gln^			
Mt4395C (T-to-C)				
Mt5821A (G-to-A)	tRNA^Cys^			
Mt7492T (C-to-T)	tRNA^Ser(UCN)^			
Mt7543G (A-to-G)	tRNA^Asp^			
Mt8343G (A-to-G)	tRNA^Lys^			
Mt10454C (T-to-C)	tRNA^Arg^			
Mt14693G (A-to-G)	tRNA^Glu^			
Mt189G (A-to-G)	H-origin	South Indian PCOS patients; only D-loop was analysed	2017	[61]
Mt310 (C7TC6-to-C8/C9/T/C6)	D-loop poly-C tract; TFAM binding site			
Mt93G (A-to-G)	D-loop	Indian PCOS patients; only D-loop was analysed *	2020	[62]
Mt143A (G-to-A)				
Mt194T (C-to-T)				
Mt205C (G-to-C)				
Mt246C (T-to-C)				
Mt447G (C-to-G)				
Mt16081G (A-to-G)				
Mt16172C (T-to-C)				
Mt16266T (C-to-T)				
Mt16270T (C-to-T)				
Mt16304C (T-to-C)				
Mt16318T (A-to-T)				
Mt16325C (T-to-C)				
Mt16352C (T-to-C)				
Mt16356C (T-to-C)				
Mt16389C (G-to-C)				
Mt16390T (G-to-T)				
Mt16524G (A-to-G)				

* Only mutations identified in 3 and more patients have been shown.

## Abbreviations

αLA	*α-*linolenic acid
ALA	α lipoic acid
AMH	anti-Müllerian hormone
AMPK	5’ AMP-activated protein kinase; 5’ adenosine monophosphate-activated protein kinase
ApoB	apolipoprotein B
ASP	acylation stimulating protein
BAX	B-cell lymphoma-2 (Bcl-2)-associated X protein
Bcl-xL	B-cell lymphoma-extra large
BMI	body mass index
CRP	C reactive protein
CVD	cardiovascular disease
DHEA	dehydroepiandrosterone
DHT	dihydrotestosterone
DM	diabetes mellitus
DPP4	dipeptidyl peptidase-4
Er	estrogen receptor
EV	estradiol valerate
FSH	follicle-stimulating hormone
GAS5	growth-arrest specific transcript 5
GH	growth hormone
GnRH	gonadotropin-releasing hormone
GPx	glutathione peroxidase
HbA1c	glycosylated haemoglobin
hCG	human chorionic gonadotropin
HDL-c	high-density lipoprotein cholesterol
HG	hyperglycemia
3β-HSD	3β-Hydroxysteroid dehydrogenase/Δ^5–4^ isomerase
IKK	IκB kinase
IL	interleukin
IR	insulin resistance
Ki-67	MKI67 (Marker Of Proliferation Ki-67)
LDHA	lactate dehydrogenase A
LDL-c	low-density lipoprotein-cholesterol
LH	luteinizing hormone
lncRNA-SRLR	(sorafenib resistance-associated lncRNA in renal cell carcinoma)
MCP-1	monocyte-chemoattractant-protein-1
MDA	malondialdehyde
MetS	metabolic syndrome
MMP	mitochondrial membrane potential
mtDNA	mitochondrial DNA
NAFLD	nonalcoholic fatty liver disease
NK	natural killer cells; or large granular lymphocytes (LGL)
NO	nitrogen oxide
OS	oxidative stress
OSI	oxidative stress index
OXPHOS	oxidative phosphorylation
PCOS	polycystic ovary syndrome
PEBP1	phosphatidylethanolamine-binding protein 1
PFK	phosphofructokinase
PI3K	phosphatidylinositol 3-kinase
PKM2	pyruvate kinase isozyme M2 isoform
PSME1	proteasome activator complex subunit 1
rRNA	ribosomal RNAs
SIRT1	silent information regulator 1; sirtuin 1
SOD	superoxide dismutase
SRA	steroid receptor RNA activator
STAT3	signal transducer and activator of transcription 3
T2DM	type 2 diabetes mellitus
TCA cycle	(tricarboxylic acid cycle); the Krebs cycle
TFAM	mitochondrial transcription factor A
Th	The T helper cells; CD4+ cells; CD4-positive cells
TNF-α	tumour necrosis factor-alpha
TOS	total oxidant status
TP	testosterone propionate
TPI1	triosephosphate isomerase
tRNA	transport RNAs
WBC	white blood cell
